# Understanding doctors’ emergency department antibiotic prescribing decisions in children with respiratory symptoms in the UK: a qualitative study

**DOI:** 10.1136/bmjopen-2021-051561

**Published:** 2021-12-20

**Authors:** Thomas Hampton, Jane Ogden, Helen Mary Higgins

**Affiliations:** 1 Faculty of Health and Life Sciences, University of Liverpool, Liverpool, UK; 2 Alder Hey Children's NHS Foundation Trust, Liverpool, UK; 3 School of Psychology, Faculty of Health and Medical Sciences, University of Surrey, Guildford, UK; 4 Department of Livestock and One Health, Institute of Infection, Veterinary and Ecological Sciences, University of Liverpool, Liverpool, UK

**Keywords:** COVID-19, respiratory infections, paediatrics, paediatric A&E and ambulatory care, paediatric thoracic medicine, qualitative research

## Abstract

**Objective:**

Exploration of the factors that influence hospital doctors’ antibiotic prescribing decisions when treating children with respiratory symptoms in UK emergency departments.

**Methods:**

A qualitative study using semistructured interviews based on a critical incident technique with 21 physicians of different grades and specialties that treat children in the UK. Interviews were audio-recorded then transcribed verbatim and analysed using thematic analysis.

**Results:**

Four themes were identified. These themes illustrate factors which influence clinician prescribing. The three principal themes were authorities, pressures and risk. The fourth transcending theme that ran through all themes was clinician awareness and complicity (‘knowing but still doing’).

**Conclusions:**

Hospital doctors prescribe antibiotics even when they know they should not. This appears to be due to the influence of those in charge or external pressures experienced while weighing up the immediate and longer term risks but clinicians do this with full insight into their actions. These findings have implications for invested parties seeking to develop future antimicrobial stewardship programmes. It is recommended that stewardship interventions acknowledge and target these themes which may in turn facilitate behaviour change and antimicrobial prescribing practice in emergency departments.

**Strengths and limitations of this study**

- This research is necessary: up to 41% of UK hospitalised children receive antibiotics and influences affecting emergency department respiratory antibiotic prescribing in particular may have a major impact on resistance.
- Despite non-random sampling, doctors from 11 hospitals contributed representing the full spectrum of seniorities and relevant specialties.
- The responses of clinicians in our interviews may not be generalisable to other instances of paediatric antibiotic prescribing or to adult respiratory patients.
- The interviewer in this study was a non-specialist, non-authority peer of the participants which may have influenced some of the responses.
- The identified responses may have been influenced by recent pandemic changes in prescribing and the resulting effect on prescribing practice and reflections.

## Introduction

Antimicrobial resistance (AMR) is recognised as a significant threat to healthcare and health systems across the world.^1 2^ Although AMR primarily concerns humans, AMR is being driven by antimicrobial exposure across healthcare, agriculture and the environment combined.^3 4^ Doctors’ antimicrobial prescriptions are common but studies have suggested that up to 50% of all antimicrobial prescriptions in the USA are unnecessary and may have a major impact driving resistance.^5^ In the UK, overall antibiotic use fell from 2013 to 2019, with primary care and dental practices (accounting for 81% of all antibiotics prescribed) making significant reductions.^6^

In contrast, hospital antibiotic use continues to climb in England rising 7.7% between 2013 and 2017.^6^ To improve understanding of the drivers and barriers to AMR and stewardship, the UK National Institute of Clinical Excellence (NICE) recommended research with prescribers that explores the reasons for antimicrobial prescribing.^7^ Currently, there are significant knowledge gaps about how to optimise antimicrobial use and resulting calls for insights in specific patient groups including children and infants.^3^

Paediatric prescribing has been identified because up to 41% of UK hospitalised children receive antibiotics.^8^ Respiratory prescribing has been targeted because antimicrobials for most respiratory tract infections are ineffective and most infections are self-limiting.^1^ Recently, COVID-19 caused widespread disruption to normal practices and greater adoption of telemedicine practices. Early clinical experiences described how in adult and paediatric settings (first published online May 2020)^9^ COVID-19 initially further disrupted appropriate prescribing of antibiotics for respiratory conditions in particular.^10^ This could be comparable with paediatric antimicrobial use in the USA, where prescriptions were made during 52% of telemedicine consultations vs 42% in hospital or 31% in primary care.^11^ Adherence to guidelines at paediatric tertiary centres for respiratory illness was poor, with paediatricians changing 93% of emergency physician prescriptions.^12^ Paediatric prescribing trends may demonstrate risk aversion but in the UK, increasing pressure to recognise and treat sepsis earlier may contribute: antimicrobial prescriptions in emergency departments (EDs) have risen 30% in the last 5 years.^2^ The notion that a well-followed guideline could prevent resistance is belied by experience^13^ and the literature assessing antimicrobial stewardship (AMS) often finds that education and awareness in clinicians have no correlation with reduced prescribing.^14^ A review in 2019 reported most AMS interventions were low quality and recommended evaluating how stewardship schemes intended to change practice.^15^ A follow-up consensus paper placed emphasis on establishing non-inferiority and a need for the possibility of individual harm to be acknowledged in study design.^16^ Decision tools rarely change prescribing, perhaps because physician prescribers can attribute social and emotional meaning to the tools, which undermines their ‘authority, autonomy and confidence’.^17^

There is a relative lack of qualitative interrogation of antimicrobial prescribing in ED settings for children with respiratory symptoms (rather than quantitative adherence to guidelines or prescribing accuracy). The present study therefore sought to explore the barriers and facilitators to antibiotic prescribing in frontline emergency and secondary care prescribers for paediatric respiratory illness specifically. The secondary aim was to assess perceptions and concerns about COVID-19 impacts on AMS.

## Methods

The study methods and reporting followed Consolidated Criteria for Reporting Qualitative research guidelines.^18^ A semistructured interview schedule was devised using a critical incident framework.^19 20^ The critical incident framework was selected for this study because it is useful to assess commonplace healthcare events. It has been demonstrated that non-confrontational recollections can precipitate critical reflections on routine practices by the interviewee, allowing not only personal development but also positive and negative reflections which contribute significantly to the analysis.^20^ Previous use of critical incident frameworks to understand clinicians’ prescribing decisions is extensively cited.^21^

Snowball methodology^22^ was used to recruit interviewees. Target recruitment was 20 clinicians using purposive sampling to achieve a spread of specialties and grades. This was based on thematic analysis literature suggesting that data saturation for novel codes usually occurs after 12 interviews and less than 4% of total codes will be determined beyond the 18th interview in a homogeneous population.^23^ Any doctor working in the UK who prescribes antibiotics to children in the ED was eligible to take part. Nurse prescribers were excluded from participation. This means that the study recruited a non-random sample of purposive participants known to the first author (doctors prescribing antibiotics in the UK to children in the ED) who after interview were invited to recommend further potential participants in the target population (who share the relevant characteristics) and who may be interested in participating in the study. There was no a priori quota sampling (ie, a mix of five consultants and five foundation doctors, etc). An initial pilot interview was used to adjust schedule prompts before finalising (interview schedule is available in online supplemental appendix 1).

10.1136/bmjopen-2021-051561.supp1 Supplementary data

After the first pilot interview, all further formal initial contacts were via email. All potential interviewees received an invitation email. If interest was expressed, an information sheet describing the purpose of the study as well as the roles and aims of the researchers was provided via email and this was reiterated in person. Participants completed consent via email before their interview. Three contacted interviewees did not participate (all three expressed initial interest and then did not respond to further contact). Interviews were conducted over Zoom (Zoom Video Communications) and audio of the interview was recorded. The interviewer was a male doctor (TH) and both interviewees and interviewer conducted interviews from private rooms in hospital or home settings, depending on time of meeting (timing of interviews was determined by availability and shift patterns of participants). Interview target duration was 20 min. Anonymised handwritten field notes were taken relating to phrasing and reception of questions only. The semistructured interview schedule was used flexibly; where topics were previously covered, prompts were omitted, adapted or expanded on, catering to each individual’s context. Participants and their interview data were anonymised using numbers. Participants’ numbers were used alongside quotes to demonstrate breadth of origin of quotations. Participants will receive email feedback on the findings after submission to a scientific journal. Participants received a certificate acknowledging their participation and the results were presented to the local respiratory team for feedback and dissemination. Recordings were transcribed using a professional transcription service (https://www.uktranscription.com) and coded using NVivo V.12 Pro software (QSR International). Transcripts were not returned to participants.

A thematic analysis of the dataset was conducted following the six-phase methodology described by Braun and Clarke.^24^ Thematic analysis is a method for identifying then analysing and reporting the themes (ie, recurring patterns) within the dataset. This process of thematic analysis requires familiarisation, code generation, searching for themes within codes, review and refinement of the themes, identifying the story that each theme tells and then reporting the data within and across the themes. None of these phases is a passive process and each researcher plays an active role in identifying the themes which they perceive. Therefore, each researcher is likely to identify and report themes which are informed by their personal experience and motivations.^25^ Thematic analysis in this study was inductive with no a priori coding framework. This means the analysis examined each interviewee’s descriptions and experiences of events and their perception of the meanings behind them rather than adjusting responses to existing discourses known to operate in healthcare interactions in our society.^24^ Although our study will have been influenced by existing relevant research in AMR,^26^ this study did not set out to prove or disprove a pre-existing hypothesis, principally because our initial literature searches revealed a dearth of previous enquiry in the specific area of antibiotic prescribing for children with respiratory symptoms in ED. Rather, the study sought to generate new data from which novel themes or understanding might be developed, based on how clinicians made sense of their own prescribing decisions in this specific context.^25^

Transcripts were read from start to finish to develop common topic ‘codes’ and then each transcript was reread and codes were combined into categories. Categories were then combined under unifying themes until no further themes were identified. After initial coding by TH, the research team discussed the identified and recurring themes until a broad understanding and consensus was achieved. HMH and JO provided contextual and interpretive insights throughout. The linear nature of the thematic phases was disrupted due to the simultaneous nature of data acquisition from the final interviews and the analysis of early transcripts. It is an understanding of thematic analysis that the iterative, inductive and reflective process of developing codes and themes will often lead to back and forth movement between phases as any study progresses.^27^ The final stage of analysis involved determination and synthesis of underlying narratives within the interviews relating to barriers and facilitators of prescribing.

### Patient and public involvement

Patients and members of the public were not involved in the design, conduct, reporting or dissemination plans of this research.

## Results

The analysis identified three major themes: authorities, pressure and risk. Transcending these was a fourth theme of ‘knowing but still doing.’ In total, 21 doctors were interviewed with a range of seniority and home specialty (see demographic data in table 1), from a total of 11 UK hospitals. All interviews took place between 1 November 2020 and 6 December 2020. Mean interview duration was 20 min 14 s (range 14:39–30:37). Data saturation in codes started to occur around analysis of the eight interview and no further interviews were planned or arranged after this time, but prearranged interviews continued until 21 were complete for consolidation.^28^

**Table 1:** Table of interviewees’ demographic data

Home specialty	Number	Region	Number	Grade	Number
Emergency	8	North West	16	Consultant	5
Paediatrics	9	London	2	ST6–ST8/senior fellow	7
GP/other	4	South West	1	ST3–ST5/junior fellow	2
		South East	2	F1–ST2	7

GP, general practitioner.

### Theme 1: authorities

Participants described the role of key authorities in terms of senior clinicians and guidelines.

#### Subtheme 1: senior clinicians

Authority figures identified by clinicians were other members of clinical staff, that is, senior colleagues or consultants from the clinician’s home specialty. These figures could directly influence practice: ‘…more senior than me, directly ordering me to prescribe… would automatically make me change my decision’ (Doctor 12).

In a few instances, the microbiology consultant or a member of the pharmacy or AMS team was identified as a positive authority: ‘the pharmacists…get in contact with you, ask you to change it…So, it works really well, I think*’* (Doctor 2).

Senior clinicians self-identified as authorities: *‘*I think we [senior clinicians or consultants] can influence the decisions…of medics that come through our department…. So, I think we can say to our trainees, “You might have been doing this elsewhere, but this is not what we do here. These are the reasons for it”’ (Doctor 14). Several consultants felt that one of the authoritative obligations was to pass on good antibiotic prescribing practices by role-modelling.

Some clinicians also identified general practitioner (GP) colleagues as figures of authority. Both junior and consultant clinicians described a desire not to undermine or contradict the prescription decisions of clinician authorities even if they did not agree with their initial prescription: *‘*If a GP started antibiotics in the community and then sends them to… [ED]…I tend to tell them to continue it. Because you’ve got one, thinking about resistance, and, two, you want to keep that patient and that parent’s trust in their GP and that relationship’ (Doctor 11).

If a GP or another doctor has previously made the decision to treat, you’re often… compelled to [continue], in a professional way. (Doctor 9)

#### Subtheme 2: guidelines

The guidelines were also perceived as sources of authority to both facilitate and withhold prescribing. Guidelines included clinical decision tools (national, specialty and local) and antimicrobial-specific guidance which many clinicians used on a daily basis: ‘The biggest help for reducing unnecessary antibiotics is clear guidelines for the presentations that could warrant them’ (Doctor 4).

Many clinicians reported only using local guidelines to decide which antimicrobial to use, once a decision to prescribe had already been made.

Senior clinicians were more likely to mention contradicting guidelines than junior clinicians, who even claimed: ‘There is a guideline and you have to do it*’* (Doctor 1). But even consultants expressed strong reservations about their prescribing: ‘A lot of the time, it’s a gut feeling, it’s a subjective decision*’* (Doctor 11).

One of our interviewees found difficulties integrating prescribing between primary care and secondary care. This did not relate to individual GPs but rather the overarching organisational guidelines of the Clinical Commissioning Group (CCG). While AMS training and teaching had been shared and delivered, the CCG guidelines were another type of authority which exerted their own low-risk decision-making processes that even a consultant could not influence.

In sum, doctors described how their antibiotic prescribing could be both improved by authorities or be less than ideal due to the influence of senior clinicians and contradictory guidelines. Many reported that *despite* guidelines, doctors recognise that antibiotic prescribing is frequently a subjective decision.

### Theme 2: pressures

Participants also described the influence of pressures with a focus on the child’s parents and restrictions due to time pressure.

#### Subtheme 1: parents

When treating paediatric patients, there is always the influence of the third party (parent or guardian), integral to the patient seeking care, expressing concerns and history taking.

‘There is always parental pressure. There are certain parents who want you to be doing something, and even though you give them the best reassurance in the world… The vast majority of these cases are caused by viral infections,…they know someone who has had antibiotics and got better. There is always a parental pressure*’* (Doctor 6). Senior clinicians were just as likely to identify parents as a pressure and experienced clinicians described how their experience and confidence in diagnosis could not always relieve pressure: *‘*This is a mild, self-limiting thing, but actually you’re not going to sleep for 10 days, and you can see the desperation in…the parents…face. You always want to give it [antibiotics] as a placebo effect’ (Doctor 14).

#### Subtheme 2: time

Time pressures were identified in two key ways to pressure and change decision-making. *‘*I must admit we were so time-pressed… I didn’t start raising questions about it*…’* (Doctor 8). Sometimes time spent in the department forced decisions before all the information was available: *‘*…end up giving an antibiotic or covering, rather [than] just give some time and see if he responds to treatment*’* (Doctor 7). Prolonged periods of time also exerted a pressure on decisions if patients either had long duration of symptoms or multiple admissions with the same condition: ‘…multiple times with the same presentation, then I feel, sometimes… that, in itself, is a huge burden to the healthcare system…so, that, sometimes, will lower my threshold for prescribing’ (Doctor 6).

Prescribing antibiotics was therefore often a result of doctors’ perceptions of external pressures including the child’s parents and time. While time pressures can be subjective, they are often objective (in busy EDs or with duration of symptoms). There is a greater potential for the subjective pressure perception of parental expectations for antibiotics not to necessarily align with parents’ actual expectations.

### Theme 3: risk

The third theme described in the data was risk which posed a discrete influence on prescribing and it became clear that the clinician’s perception of risk was a key driver in many of the social interactions described both generically and also during the period of uncertainty which occurred early during COVID-19.

#### Subtheme 1: generic risk

The impact of risk was principally to cause ‘just-in-case’ prescribing among both junior and senior doctors: ‘I am aware that it’s not just a risk-free decision*’* (Doctor 12). This could be mitigated by relationships described between senior doctors and parents, and also with their level of comfort or confidence in deviating from guidelines or withholding antibiotics: *‘*I think the difficulty is that paediatrics is quite a grey area…. You ask 10 paediatricians, “Would you prescribe antibiotics?” you might get a split answer. You know, eight might, two might not, or the other way around… it’s about the safety-netting*’* (Doctor 11). There were a number of references to risky decisions. Perception of this risk appeared to change with increasing complexity, acknowledging that prescribing decisions can change due to the increased risk for children in certain specific circumstances: ‘in the clinical setting of … transplant… or potential immuno-compromise… our children go home on prophylactic antibiotic and that is just accepted*’* (Doctor 20). This extended to children living with diabetes, sickle cell, Down syndrome, cystic fibrosis or any neonates: *‘*So if it’s a teeny one [baby] or the patients that are chronic or have lines in… I wouldn’t be too brave…personally*’* (Doctor 7).

#### Subtheme 2: COVID-19 risks

COVID-19 provided a useful prompt to encourage insight into clinician’s context-dependent subjectivity. Several clinicians cited the ability to use chest X-rays to distinguish between viral and bacterial chest infections but admitted that COVID-19 X-rays looked like ‘consolidation type X-rays*’* (Doctor 1). Many admitted that investigations may not change management: *‘*we tend not to X-ray children…the outcome will be antibiotics anyway*’* (Doctor 4), and the need to manage uncertainty in the new paradigms of COVID-19 allowed many to acknowledge previous non-objective risk management: ‘you’re basing your judgement on more clinical and history factors than actual objective evidence*’* (Doctor 14).

Some participants reported increased antibiotic prescribing earlier in the pandemic (particularly when they also worked in adult settings): *‘*Anyone now who has got a slightly prolonged fever is getting antibiotics… I’m probably less resistant now. I think, you know, I used to be really, really, resistant to giving antibiotics*’* (Doctor 17).

However, many clinicians felt their prescribing either did not change at all, or had now returned to pre-COVID-19 levels. Some reported improved behaviours as part of their experiences and reflections on previous practice: *‘*I think, during COVID-19, it was probably easier to get across the idea of viral upper respiratory infection. Because everybody had heard of coronavirus and it’s got ‘virus’ in the name. So, antibiotics won’t work. And people would suddenly be like, “Oh yes, I know that”*’* (Doctor 1).

Perceptions of both generic risks and COVID-19-specific risks influenced the participants’ prescribing of antibiotics. COVID-19 did not necessarily reveal novel prescribing practices but instead acted as yet another risk factor which illuminated many existing pressures regularly exerted on the decision-makers. Ultimately, the decision-making among the interviewed clinicians depended on how each individual reconciled the balance between authorities, pressures and risks.

### The transcending theme: ‘knowing but still doing’ or acknowledgement and insight to tensions and complicity

Participants described how their prescribing was influenced by authority, external pressures and a perception and balance of immediate and longer term risk. This created a sense of cognitive dissonance in decision-making. There were tensions, negotiation and need for resolutions between barriers and facilitators of antimicrobial prescribing and many agreed that their decision-making was not necessarily reproducible or evidence based. From the transcripts, there was an over-riding sense that all the doctors felt that when prescriptions were made that might not be indicated, this was a conscious, deliberate decision made in full acknowledgement of the conflicts with their preference: ‘Sometimes because of the way you take the sample…with lots of squeezing [of the finger during finger prick blood tests], it will come back with a high lactate… this then flags as septic [referencing sepsis 6 scoring tool—a type of guideline]…then you’re in a position where you can’t really go back up the path and say you think it’s a minor viral illness*’* (Doctor 1). Final decisions to start or stop antibiotics essentially did not always depend on the actual clinical findings; they were made ‘….depending on the senior doctor you were to ask*’* (Doctor 1).

Initially, many doctors suggested that strict criteria were required to prescribe antibiotics: *‘*In general I would be loathe to prescribe antibiotics*’* (Doctor 19). However, during the interview, nearly all doctors conceded that these criteria are subject to interpretation: *‘*The problem is that you just can’t tell without swabbing, or without testing*’* (Doctor 14), and highly context dependent. Senior doctors tended to acknowledge that antibiotics were sometimes initiated as part of a larger group of interventions when children were ‘sicker’ regardless of the diagnosis: ‘you know, if they’re sick enough to need non-invasive ventilation then surely, they must need antibiotics?*’* (Doctor 17). Doctors acknowledged a lack of feedback to inform decision-making, in particular for emergency patients who get sent home with oral antibiotics but never return to the department: ‘it might have just been that they were going to get better by themselves anyway…’ (Doctor 14).

More emergency physicians acknowledged a future-discounting bias whereby they achieve resolution in times of uncertainty by taking an interventionist decision to treat the individual in front of them (as befits their job role, rather than thinking long term about the population as a whole or AMR): *‘*I feel like, you know, our stress is to make sure the right people are getting antibiotics quickly and not more about long-term unnecessary use’ (Doctor 5). Some physicians identified the need to assent to parental demands to maintain a relationship as the first step in their child’s treatment: *‘*inhalers or antibiotics … admission….or whatever….if it means that they feel a bit more reassured and it changes the dynamics slightly with their parenting, then that’s absolutely [done- (the antibiotics are given)]—No evidence-based whatsoever, it’s purely from an emotional standpoint. I’m saying it out loud like a recording… of their anxiety, or parental experience*’* (Doctor 14).

Two clinicians were openly worried that their trusts had room for improvement with antibiotic stewardship; they reported knowing but were still doing it anyway. Very few felt that GPs or other specialties should take responsibility for resistance: ‘we use way more antibiotics in bronchiolitis and viral wheeze…than anywhere else I’ve worked!’ (Doctor 17).

## Discussion

This study identifies key social determinants that influence antibiotic prescribing by clinicians treating children with respiratory symptoms in ED. The major themes were authorities, pressures and risk, with a transcending theme of ‘knowing but still doing’. This study’s findings predominantly agree with existing qualitative research on influences on antibiotic prescribing for respiratory conditions (namely, balancing the perception of authorities, guidelines and risk).

Both authorities and pressures could be perceived as barriers or facilitators to antibiotic prescribing depending on context. The identified risks (of harm) usually led to prescribing (rather than avoidance of prescribing to minimise risks of resistance) for a child. Managing unwell paediatric patients can be frightening for all involved as many children are difficult to assess and may be more unwell than they appear.^29^

Identifying senior clinicians as authorities parallels the findings of Papoutsi *et al*^30^ and Davari *et al*.^31^ Davari *et al* also identified diagnostic and antimicrobial guidelines as a facilitator of prescribing.^31^ Time pressures (including a need to make choices without all the information) and the burden of prolonged time, for example, of fevers or repeated presentations, inclined clinicians to prescribe but the expectations of parents could be a barrier or facilitator. Each clinician’s perception of risk attached to their decisions often related to complexity of the case and generic examples drew parallels to specific uncertainty during COVID-19. The clinician authority’s influence on practice depended on their personal antibiotic prescribing and risk behaviours, and the relationships, identity and roles of the prescribing clinician within that authority’s department. Previous reports of cultural and intrinsic subspecialty differences driving prescribing differences^32^ were not obviously apparent in this study. All clinicians were asked about identical patients in identical contexts and there may be some paediatric patients with specific presentations that would only be treated by one specialty if a prospective, real-world pragmatic study was performed. This study supports existing work documenting the influence of hierarchy and authority on lifelong prescribing habits among junior doctors.^33^ But this fails to acknowledge that senior doctors themselves also operate in complex social, multidisciplinary environments and may be subject to similar authorities and pressures themselves.^34^ This study also found clinicians working in the ED (regardless of specialty) exhibited ‘future discounting’ (thinking principally about risks ‘in the here-and-now’) when thinking about risk, which has also been well researched. Managing *‘*immediate clinical risks, reputation and concordance with peer practice*’*^35^ seems to far outweigh longer term consequences in the context of the hospital and hospital practice.

Many of the determinants identified in this study draw parallels with attempts to map the complexities of AMS to a conceptual framework.^36^ This involves incorporating the wider healthcare system organisation (including interactions between medical juniors, hospital consultants, GPs and community team members) with the specifics of the social interactions between patients and clinicians and the underlying cultural beliefs of all parties. These determinants and barriers also map to realist programme theories.^30^ Some of the mechanisms identified here can be conceptualised as clinicians managing ‘their own risk’. Awareness and complicity can manage risk or fear of criticism and the risk of individual responsibility and culpability. Clinicians’ reputations can be put at risk if decision-making does not follow fashion or convention in that hospital. NICE’s guidelines on AMS found that barriers tended to fall into: clinical priorities, decision-making, hierarchies and resources (with obvious parallels to risks, tensions, authorities and pressure).^37^ Some of the guidelines identified could be conceptually graded by influence and strength of authority. Specific society guidelines (British Thoracic Society) generally held more sway than national (‘NICE’) guidelines and trust-specific antimicrobial policies, although trust-specific guidelines were the most frequently mentioned. Among experienced clinicians, that is, emergency medicine consultants without a direct senior clinician, it was reported that knowledgeable parents of complex patients are essentially ‘promoted’ to additional authorities (who continue to exert a perceived pressure). These clinicians had the clearest understanding that their antimicrobial prescribing was not necessarily symptom, investigation or evidence-based but was nonetheless seen as necessary to maintain a relationship with that parental authority. There is ample evidence to suggest the social and behavioural aspects of clinicians are frequently overlooked in the design and implementation of AMS interventions.^32^ Many quality improvement initiatives or complex interventions in this area fail to make lasting change due to adaptive challenges.^38^ To better enact meaningful change in the future, this study aimed to identify the external forces which might reveal the motivations and intentions of the specific clinicians whose prescribing behaviour would be the target of such behaviour change initiatives.^38^ Future studies in this field are advised to replicate this study’s thematic approach so that both datasets’ identified themes can be compared using a structured verification of each of the six phases of the analysis.^27^

There were two key areas where this study’s findings contrasted with the broader literature on the topic and previous qualitative findings. One was the relative lack of confrontational tribalism between specialties, with an uncharacteristic openness about personal or departmental overprescribing in circumstances where it may not be indicated. This study identified no clinicians resorting to previously cited exceptionalism to exonerate prescribing behaviour, the most common being to attribute each clinician’s own patients as somehow ‘being special’.^39^ The second contrary finding was the clear narrative from the dataset that all the clinicians knew (or at least retrospectively acknowledged) that decisions are regularly made which do not follow best practice. It is often cited during AMR research that the problem of resistance and adherence to prescribing is significant in other specialties or regions compared with the clinicians being surveyed locally^40^ but this study conflicted with that trend. There was openness about the deliberate tension between knowledge and action rather than a suggestion that education is lacking and that knowledge gaps or complexities were forcing these decisions to be made in unintentional error.^41^

### Limitations

This study was limited by the number of UK hospitals from which antibiotic prescribers were recruited, the non-random recruitment and the lack of nurse prescriber recruitment. Nonetheless, physician participants of varied experience and rank contributed from 11 hospitals with wide geographical spread across England. While the influence of COVID-19 may offer unique insight into that specific time period, some of the comments may therefore be less generalisable to antibiotic prescriptions for children at other times. Despite an attempt to analyse themes without an a priori coding framework, it is unlikely that the positionality of the researchers allowed existing assumptions about AMR to be completely excluded from their interpretation of the transcripts. The strength of the research team is that three different viewpoints were brought: with psychological, veterinary and medical lenses. Nonetheless, the interviewer was a male medical hospital doctor and this may have had an additional impact on the participants’ responses and dynamic during interviews, as well as the framing of the conclusions. It is worth clarifying that the interviewer was not a respiratory or microbiological specialist and had no hierarchical influence over any of the participants identified, although the research team’s prior exposure to Global Health and One Health may impact on both participant recruitment and responses. Multidisciplinary collaboration with inclusion of AMS champions who are peers, that is, someone considered to be from their own discipline, has previously demonstrated utility in AMS interventions^42^ and some of the insights gained from this study may in part relate to the interviewer’s existing relationships or a collegiate sense of similarity among doctors with a researcher they may not see as a direct authoritative challenge to existing practices.

## Conclusions

What can researchers and clinicians do to combat the influence of the major themes identified here when there is also an acknowledgement of the transcending theme of complicity or ‘knowing but still doing’ that clearly underpins existing prescribing habits? One suggestion was to consider rebranding viruses; with COVID-19 as a representative virus that the public perceives as a serious pathology, but still one that does not need antibiotics. This pandemic could be a chance to change people’s perceptions of being ‘fobbed off’ or feelings that their symptoms are not taken seriously when they do not receive antibiotics. A new intervention should consider psychosocial implementation theories^43^ or even a quality improvement lens to further explore adherence to antibiotic guidelines. Adoption and change barriers might be identified among self-aware clinicians if future qualitative studies consider the potential for the clinician to perceive both ‘real’ (time or autonomy) and ‘perceived’ losses (loss of their autonomy, decreased power, increased workload).^38^ This study demonstrates some of the key social determinants of antibiotic prescribing in a specific context of children with respiratory symptoms in ED. The results should inform the design and development of future stewardship schemes hoping to impact on AMR in children.

## Data Availability

Data are available upon reasonable request. The original transcriptions generated during the current study are not publicly available due to confidential and anonymous nature of the recording contents but other data from this study are available from the corresponding author on reasonable request.
