# Supplementary material for: Understanding doctors’ emergency department antibiotic prescribing decisions in children with respiratory symptoms in the UK: a qualitative study
Source: BMJ Open. 2021 Dec 20;11(12):e051561. doi: 10.1136/bmjopen-2021-051561 (PMC8688728; doi:10.1136/bmjopen-2021-051561)
Supplement: Supplementary data [file bmjopen-2021-051561supp001.pdf]

APPENDIX 1

INTERVIEW SCHEDULE

A Opening questions

This interview is trying to establish how antibiotic prescribing habits might have been impacted by covid. Firstly I'd like to ask you about your professional experience to put your answers into context.

- Where do you work now and how long for?
- How often do you treat children (age 15 and under)? Do you also treat adults (16+)?
- Have you worked elsewhere treating children before?

B Main questions about Antibiotic use

Thank you.

Now I'd like to talk about your role in prescribing antibiotics for the children you treat in your current role.

[Critical Incident 1]:

1.1 Please can you tell me about a common-place patient with respiratory symptoms when your management included antibiotics?

1.2 Why did you decide to prescribe/use antibiotics on this occasion?

1.3 What made you think this was the best approach to take here?

[Critical Incident 2]:

2.1 Ok, please can you tell me about another example of when you had to give antibiotics for a respiratory condition, where the decision was more/less clear-cut?

2.2 Why did you decide to prescribe/use antibiotics on this occasion?

2.3 What made you think this what the best approach to take here?

[Critical Incident 3]:

3.1 Can you tell me about a recent example when you could have given antibiotics for a respiratory condition but you decided not to? (prompt: eg croup if no suggestion)

3.2 Why did you decide NOT to prescribe/use antibiotics on this occasion?

3.3 What made you think about best practice here?

[Critical Incident 4]:

4.1 Please can you tell me of any examples when you gave antibiotics in a different way during the coronavirus pandemic when you may not have otherwise?

4.2 Why did you decide to prescribe/use antibiotics on this occasion?

4.3 What were your thoughts about best practice to take here?

[Additional questions…Asked only if these topics have not been touched on during critical incident discussions]

- Are there any other factors that **influence**/pressurize your decisions to prescribe/use antibiotics?
- (consultant only-Do you ever consider using antibiotics **off-label** (prompt i.e. Azithromycin if denied)?)
    - Why / Why not?
- Do you use any specific **guidelines** when you decide to prescribe/use antibiotics?
- Do you ever speak to parents about the **amount** of antibiotics their children are receiving?
- Do you ever speak with parents or children about **how they should use** antibiotics, and why?
- How **comfortable** are you with current levels of antibiotics used for your patients?
    - Does the hospital you are in change your prescribing? (i.e. tertiary centre)
    - Does the team you are in change your prescribing? (i.e. resp versus ED)
    - Does the dynamic with the parents change your prescribing?
- Do you have any other comments about antibiotic prescribing/use, either from your own experience in this hospital, or in general? ?mention seasonality?

C Main questions about antimicrobial resistance (AMR) beliefs

Ok, so now I'd like to ask you about antimicrobial resistance as I'm interested in your thoughts and opinions on this topic.

1.1 Does the issue of AMR influence your management?

1.2 Do you think about the consequences of AMR in your daily practice?

I'd like to ask you some questions now about AMR, that will relate firstly to children and then to their parents/guardians

2.1 Do you think that paediatric rates of antimicrobial resistance are changing? (prompt: increasing, decreasing or stable?)

2.2 Do you think antibiotics in hospital or primary care has a greater role in driving resistance?

2.3 What role, or influence do you think parents and guardians have to play in addressing the issue of AMR?

2.4 What role, if any, do you think paediatricians/doctors have to play in addressing the issue of AMR/ stewardship?

2.5 How concerned would you say you were/are about antibiotic stewardship during the covid-19 pandemic vs previously?

2.6 And how do you feel about it in your practice right now?

2.7 Have you ever thought about antibiotic resistance in animals and farming, and whether this might have an impact on humans?

 [Prompt if simple Yes/No answer] – Please can you explain why you think that?

D Closing remarks

3.1 Suggestions for the future

- What practical initiatives might help you or your colleagues reduce/optimize the amount of antibiotics you prescribe for children?
- Do you have any other comments about antimicrobial resistance?

Thank you for your time. Are there any additional comments you would like to add?
